# Hemo-Lymphopoietic Malignancies Surround the Women of the Family: A Case Report and Literature Review

**Published:** 2018-04-01

**Authors:** Behjat Kalantari-Khandani, Ali Akbar Haghdoost, Mohsen Momeni, Mina Danaei

**Affiliations:** 1 Department of Oncology, Kerman University of Medical Sciences, Kerman, Iran; 2 HIV/STI Surveillance Research Center, and WHO Collaborating Center for HIV Surveillance, Institute for Futures Studies in Health, Kerman University of Medical Sciences, Kerman, Iran; 3 Modeling in Health Research Center, Institute for Futures Studies in Health, Kerman University of Medical Sciences, Kerman, Iran; 4 Social Determinants of Health Research Center, Institute for Futures Studies in Health, Kerman University of Medical Sciences, Kerman, Iran

**Keywords:** Leukemia, Lymphoma, Neoplasms, Risk factors

## Abstract

The co-occurrence of different types of hemo-lymphopoietic malignancies within a family provides clues about the pattern of inheritance and common environmental risk factors. A family presented with developing hemo-lymphopoietic cancers in three female first-degree relatives: a mother and her daughters.

Case 1 was diagnosed with Walden Strom's macroglobulinemia at age 57. Case 2 and 3 presented with chronic myelogenous leukemia at age 32 and diffuse large B-cell lymphoma at age 28, respectively. There were not any significant common environmental risk factors in this family, but all three cases suffered from skin dermatitis and one of them, who suffered from chronic myelogenous leukemia, was diagnosed with morphea. This family had a sedentary and stressful lifestyle.

Genetic is the foundation of some familial aggregation of cancers. Common lifestyle habits and environmental etiologies are important. Morphea as an autoimmune disease could have the essential role in developing hematolymphoid malignancies.

## Introduction

 Today, malignancy is one of the major health problems in the world. It is estimated that the number of new cases of malignancy will reach to 15 million per year in 2020 and approximately 60% of these new cases will be from developing countries ^1^. In Iran, the incidence of malignancy is nearly 98 to 110 cases per 100,000 population and cancer is the third leading cause of death^   2 ^. Excluding skin cancers, lymphoma and leukemia are among top 10 cancers in Iran^3^.

Family history and exposure to the carcinogenic agents are important in cancer development ^4^. Familial aggregation of cancer provides clues about the malignancy etiology, the pattern of inheritance and the probable common environmental exposures ^5^^, ^^6^ .Different studies indicated the familial cluster of Non-Hodgkin lymphoma (NHL) and other hematolymphoid (HLP)tumors ^5^^, ^^7^^-^^11^. The relative risk of NHL / HLP cancers was estimated 1.5 to 4 fold in relatives of NHL patients in epidemiologic studies^   12 ^. Two case reports declared the occurrence of NHL in three brothers and also in a father and his son, respectively ^7^^, ^^8^ .

To the best of our knowledge, the majority of familial aggregation of HLP malignancies and different types of Lymphoma, especially NHL, have been reported in men relatives. In this paper, we report an exceptional case of familial aggregation of HLP cancers among all women in a family. There were three cases of hemo-lymphopoietic cancers in the first-degree relatives, a mother and her daughters diagnosed within a one-year period. Moreover, we reviewed the related literature and discussed the potential mechanism of this rare occurrence.

## Case presentation

**Case 1**

A 57-year-old housewife (mother of a family) suffering from weakness and fatigue was admitted into our clinic. The results of cell blood count (CBC) showed Hb=5 normochromic normocytic anemia. The level of ferritin was 394 Nano grams per milliliter. The level of total bilirubin and direct bilirubin were 4.3 and 0.8 milligrams per deciliter, respectively. The level of LDH was 800 milligram per deciliter. Direct and indirect coombs tests were strongly positive. According to the results of bone marrow biopsy and immunoelectrophoresis, the definitive diagnosis of Waldenstrom's macroglobulinemia (WM) was made. She was treated with a combination of rituximab, cyclophosphamide, dexamethasone and thalidomide. She did not experience relapse episode.

She had five children; two daughters and three sons. She used to suffer from dermatitis and eczema many years ago. There was not any other significant chronic disease or immunodeficiency disorder in her past medical history. In the past health history, regardless of solid fats in her diet, she maintained a well-balanced diet, but she had a sedentary lifestyle with stress. Her body mass index was normal. She used mobile phone rarely. Regarding the social determinants of health, she came from a family of the middle socioeconomic status and lived in a crowded neighborhood. In her family and personal history, there were not any significant familial or environmental risk factors for lymphoproliferative cancers including carcinogenic drugs, pesticides, herbicides, hair color, air freshener spray, detergents, tobacco or other chemical carcinogenic agents.

**Case 2**

A 32-year-old married housewife, who was the fourth child of her family, complained that she had been suffering from fatigue and abdominal pain for the past 15 years. The spleen was palpable 3 cm below the costal margin. The following results were noted: WBC: 100,000, Hb: 10, and PLT: 800,000. The level of MCV, MCH and MCHC were within normal range. The results of bone marrow aspiration and biopsy confirmed the chronic myelogenous leukemia (CML). She was treated with 400 milligrams of imatinib per day and responded to the treatment. She did not experience any relapse.

She had a daughter and a son. In her past medical history, she had been suffering from morphea-like patches on her abdomen and back (a localized type of scleroderma that causes discolored, painless patches on the skin) for the past 15 years. Her healthy brother has also experienced morphea. She was a stressful person and had not enough physical activity, but followed a healthy diet. The patient was considered obese with a BMI at nearly 30. She played computer games on her own cell phone at least 2 hours daily and kept it close by overnight. She had been living in the same neighborhood as her parents until 3 years before diagnosis of her malignancy. No significant environmental risk factor was detected in her personal history.

**Case3**

The fifth child was a 28-year-old married housewife suffering from fever and abdominal pain. She had splenomegaly without systematic lymphadenopathy. Findings of sonography showed two hypoechoic masses (10×8 and 29×27 millimeter). The results of abdominal and pelvic computed tomography (CT) with oral and intravenous contrast were similar to the sonography findings. There were not any abnormal findings in lung CT scan and bone marrow biopsy. Splenectomy was done and splenic involvement by diffuse large B-cell lymphoma was identified. Following surgery, the patient received 8 cycles of R-CHOP chemotherapy (Rituximab: 375 mg/m^2^, Cyclophosphamide: 750 mg/m^2^, Doxorubicin: 50 mg/m^2^, Vincristine: 1/4 mg/m^2^, Prednisone: 100 mg/m^2^). Meanwhile, 8 cycles of Rituximab were also administered every three months. Finally, the patient entered remission and has not experienced any relapse until now. Her past medical history showed that she suffered from infertility and was childless. The past health history was the same as other family members. Her body mass index was normal. She did not spend more than an hour on her mobile phone daily. She lived in the same neighborhood as her parents. The patient did not report over exposure to the environmental risk factors. The descriptive characteristics of all three cases are presented in Table1.

**Table 1:** Descriptive characteristics of three cases

**Demographic ** **characteristics**		**Chief ** **complaint**	**Diagnoses**	**Risk factors**		
**Age **	**Occupation**			**Lifestyle**	**Socioeconomic**	**Medical history**
57 years	Housewife	-Weakness -Fatigue	WM*	-Consuming solid oils -Sedentary lifestyle -stressful lifestyle	-Having middle socioeconomic status -Living in crowded neighborhood	Dermatitis and eczema
32 years	Housewife	-Fatigue -Abdominal pain	CML**	-Consuming solid oils -Sedentary lifestyle -stressful lifestyle -Obesity Mobile phone overuse	-Having middle socioeconomic status -Living in crowded neighborhood	Morphea
28 years	Housewife	-Fever -Abdominal pain	DLBCL***	-Consuming solid oils -Sedentary lifestyle -stressful lifestyle	-Having middle socioeconomic status -Living in crowded neighborhood	Infertility

* Walden Strom's macroglobulinemia,

** Chronic myelogenous leukemia,

*** Diffuse large B-cell lymphoma

## Discussion

 It has been estimated that hematologic cancers are approximately 3 times more frequent in families with history of hematologic malignancy than other families^   8 ^. Moreover, the lifetime risk of developing NHL in the first degree relatives of NHL patients (3.6%) is higher than the population risk (2.1%), especially in those with early or late onset NHL disease ^6^^, ^^7^ . Some case-control and population-based studies have investigated the risk of familial aggregation of hematopoietic and lymphoid cancers. Table 2 and 3 summarize the results of each study. Some fluctuations in the results could be explained by different sample sizes and different indicators investigated in each study. Overall, family history of different types of hematopoietic and lymphoid cancers was a significant risk factor for hemo-lymphopoietic cancer development ^5^^, ^^6^^, ^^9^^, ^^10^^, ^^12^^-^^16^. 

In this report, blood cancer and lymph node metastases were found in the female members of a family. One report demonstrated the familial aggregation of lymphoid malignancies (NHL) in two male members of a family; father and his son. During a 6-month period, both were diagnosed with cancer^6^. Another report demonstrated a familial cluster of NHL in 3 brothers at the age of 45, 52 and 56 years, respectively. They were all cigarette smokers and had exposure to the carcinogenic agents in their workplace^8^. 

**Table2:** Description of population-based studies about the familial risk for Hemolymphopoietic malignancies

**First Author**	**Publication ** **Year**	**Index case**	**Non-Hodgkin ** **Lymphoma**	**Hodgkin Lymphoma**	**Leukemia**	**Hematopoietic ** **cancer**
			**Risk among first-degree relatives (RR/SIR, 95% CI)**			
Kristinsson, S. Y*	2008	LPL/WM	3(2-4.4)	0.8(0.3-2.2)	3.4(1.7-6.6)**	-
						
Paltiel, O***	2000	LPL	1.7(0.9-2.9)	1.2(0.4-2.5)	1.1(0.4-2.3)	1.4(0.9-1.9)
						
Paltiel, O***	2000	NHL/HL	1.7(0.9-2.9)	1.2(0.4-2.5)	-	-

* **: Relative Risk,**

** **: Only CLL,**

*** **SIR**

**Table3:** Description of case-control studies about the risk of NHL or HL by family history of NHL, HL, Leukemia and hematopoietic cancers

**First Author**	**Sample size**	**Publication ** **Year**	**Type of ** **lymphoma in ** **cases**	**Non-Hodgkin ** **Lymphoma**	**Hodgkin ** **Lymphoma**	**Leukemia**	**Hematopoietic cancer**
				**Family history (OR,95% CI)**			
Wang,S.S	10211 cases& 11905 controls	2007	NHL	1.5 (1.2-1.9)	1.6 (1.1-2.3)	1.4 (1.2-2.7)	1.4 (1.2-1.6)
Mensah, FK	699cases& 742controls	2007	NHL	-	-	1.4 0.8-2.6)	1.7 (1.1-2.7)
Negri,E	225cases& 504 controls	2006	NHL	1.8 (0.3-9.7)	-	2 (0.6-5.9)	3 (1.2-7)
Casey, R	2480cases& 2540controls	2006	LN	-	3.4 (1.5-7.8)	1.5 (1.1-2.1)	1.6 (1.2-2.1)
Chang, E.T	1506 cases&1229 controls	2005	NHL	2.2 (1.4-3.5)	0.4 (0.1-1.9)	3.2 (1.2-8.8)*	1.8 (1.2-2.5)
Chang, E.T	1506 cases&1229 controls	2005	HL	3.3 (1.3-8)	3.3 (0.5-21.5)	6.3 (1.3-9.9)*	2.1 (1-4.3)
Chatterjee, N	1321cases& 2409controls	2004	NHL	2.06 (0.73-5.76)	1.6 (0.5-5.1)	1.1 (0.6-2.1)	1.2 (0.8-1.8)
Goldin, L.R	5047cases& 10078controls	2004	HL	1.26 (0.9-1.75)	3.11 (1.82-5.29)	2.11 (1.18-.8)*	1.65 (1.3-2.1)

* **: Only CLL**

A case-control study with a large sample size demonstrated that the risk of NHL is higher in a person having a sibling with NHL (OR: 2, 95%CI: 1.4-2.8) than parents with NHL (OR: 1.4, 95%CI: 1.0 -1.8). NHL in the male members of a family was a greater risk factor for both men and women relatives (OR: 2.1, 95%CI: 1.5-3.0) rather than female members (OR: 1.3, 95%CI: 1.6-4.8). Having a brother with NHL was the strongest risk factor for both women and men(OR: 2.8, 95%CI: 1.5-3.0) rather than having a father with NHL(OR: 1.8, 95%CI: 1.1-2.9) ^9^.Another case-control study estimated the hazard ratio of developing hemo-lymphopoietic cancers in the first-degree families and showed significantly increased risks of hemo-lymphopoietic cancers, especially in female relatives. In contrast to NHL, the risk of hemo-lymphopoietic malignancies was higher in siblings (HR: 5.3, 95%CI: 1.4-16.8) than parent-offspring pairs (HR: 2.3, 95%CI: 0.8-6.5)^12^. Findings of the previous studies demonstrated that individuals having a sibling with leukemia are at higher risk of developing NHL (OR: 1.7, 95%CI: 1.3-2.2) than those having a parent with leukemia (OR: 1.4, 95%CI: 1.1-1.7). NHL was more common in women who had a sibling with leukemia (OR: 2.2, 95%CI: 1.5-3.2), especially who had a sister with leukemia(OR: 3, 95%CI: 1.6-5.6) ^9^. These findings could explain the simultaneous occurrence of CML and NHL in two sisters and the aggregation of NHL in mother and her daughter.

Twin studies have demonstrated that the risk of lymphoma in monozygotic tween is more likely than dizygotic ones. There is an association between the risk of lymphoma and polymorphism in immunity and inflammation genes, DNA repairman genes and genes linked to the metabolism of folate ^   17 ^. Genetic polymorphisms could have an important role in a familial cluster of NHL    9 .Parents of this family had a consanguineous marriage. The importance of genetic transformation was highlighted in these cases.

In this report, the woman with CML and her brother had a history of morphea (an autoimmune disease) in the past. The mother of the family also had skin lesions and dermatitis many years ago. Sometimes, familial lymphoma has been linked to the congenital immune deficiency syndrome ^17^. Two case reports demonstrated the association between morphea (circumscribed scleroderma) and lymphoma^18^^, ^^19^. To the best of our knowledge, there were not enough studies about the association between hematopoietic malignancies and morphea, but the current study raises the concern about it.

On the other hand, there are some biological and environmental risk factors for lymphoma including viral infections (EBV, HTLV-1, and HIV), history of chemotherapy and radiation, history of exposing to solvents, pesticides and herbicides ^17^. Cancer in families suggests the basic role of genetic and the predisposing role of early environmental exposure. In some studies, there was a significant interaction between the genetic and environmental factors, including infectious agents, pesticides and herbicides, dietary habits, radiation, tobacco, blood transfusion, occupational exposure and using some drugs, but in some others there were no significant differences in known causal environmental factors between the two groups with and without NHL family history. In addition, environmental factors had only a marginal effect in these studies^9^^, ^^10^. There was no significant report of environmental exposure in these cases explaining the aggregation of cancer in this family. Some familial aggregation of cancer could develop only by chance. It seems that genetic and chance are two determinant factors for clustering cancer in this family.

This study is unique because in this family, against most of the studies, only female members presented the malignancy, and three different types of WM, NHL, and CML were presented in different members in a short period of time. These family members did not expose to the major known environmental risk factors and they had only a personal and family history of skin disorders. Further studies are needed to determine whether there is an association between morphea and hemo-lymphopoietic cancers.
